# An updated job-exposure matrix for occupational noise: development and validation

**DOI:** 10.1093/annweh/wxad074

**Published:** 2023-12-09

**Authors:** Mattias Sjöström, Marie Lewné, Magnus Alderling, Jenny Selander, Per Gustavsson

**Affiliations:** Institute of Environmental Medicine, Karolinska Institutet, Solnavägen 4, 10th floor, SE 113 65 Stockholm, Sweden; Centre for Occupational and Environmental Medicine, Region Stockholm, Solnavägen 4, 10th floor, SE 113 65 Stockholm, Sweden; Institute of Environmental Medicine, Karolinska Institutet, Solnavägen 4, 10th floor, SE 113 65 Stockholm, Sweden; Institute of Environmental Medicine, Karolinska Institutet, Solnavägen 4, 10th floor, SE 113 65 Stockholm, Sweden; Institute of Environmental Medicine, Karolinska Institutet, Solnavägen 4, 10th floor, SE 113 65 Stockholm, Sweden; Institute of Environmental Medicine, Karolinska Institutet, Solnavägen 4, 10th floor, SE 113 65 Stockholm, Sweden; Centre for Occupational and Environmental Medicine, Region Stockholm, Solnavägen 4, 10th floor, SE 113 65 Stockholm, Sweden

**Keywords:** Job exposure matrix (JEM), occupational noise

## Abstract

**Objectives:**

The aim of this study was to create a quantitative job-exposure matrix (JEM) for noise including a large set of measurements for the Swedish workforce, a detailed exposure-level assessment, spanning over an extensive time period from 1970 to 2014.

**Methods:**

The JEM was developed by 2 teams, each with an experienced occupational hygienist and an occupational safety engineer. Each pair assessed the exposure using measurements performed and reported by occupational hygienists, occupational safety engineers, or similar, from 1970 to 2014. The measurements included either the original LAeq(8h) measurements or an LAeq(8h) levels calculated from partial measurements of the working day, provided that the measurement targeted a regular task usually performed during a full workday. The collection of measurement reports was done in 2008 and 2012 by contacting clinics working in the area of occupational health or occupational safety engineers and their submitted reports were added to our own material. Noise exposure assessments were inserted at the appropriate time period for the relevant job family. The final matrix was developed in a consensus procedure and the validity was investigated by comparison of the 2 team’s individual results.

**Results:**

The noise JEM contains 321 job families with information regarding occupational noise from 1970 to 2014. The time-period label has a 5-yr scale starting in 1970. The estimated average 8 h (TWA) noise level in decibels [dB(A)] for every job family and 5-yr period was coded as 1: <70 dB(A), 2: 70 to 74 dB(A), 3: 75 to 79 dB(A), 4: 80 to 84 dB(A) or 5: 85(+) dB(A). The validation showed no systematic difference in relative position and very high agreement in the ordering of paired ordinal classifications. The JEM has also successfully been applied in several epidemiological studies.

**Conclusions:**

We present a JEM for occupational noise using Swedish data from 1970 to 2014 with a higher degree of sensitivity in assessed noise exposure compared with the previously existing version. Repeated application of the JEM, in epidemiological studies, has shown consistent results and contributed to yielding important findings.

**What’s Important About This Paper?**

In this study, a new job-exposure matrix for noise was constructed based on a large set of noise measurements for the Swedish workforce over a period of 44 yr and expert assessments. The matrix assigns 5 categories of 8-h time-weighted average noise levels to every job family for 5-yr periods. The matrix was validated and applied in several studies yielding consistent and reliable results, enabling new epidemiologic analyses.

## Introduction

Noise is a well-known and common occupational exposure that affects millions of workers worldwide (Tak et al. 2009; Tikka et al. 2017). Hearing impairment (Lie et al. 2016; Sturman et al. 2018; Fredriksson et al. 2021) is the most studied outcome of noise exposure but other effects have also been reported such as increased blood pressure, ischemic heart disease (Theorell et al. 2016; Eriksson et al. 2018), increased risk of myocardial infarction (Davies et al. 2005; Virkkunen et al. 2005; Selander et al. 2013; Skogstad et al. 2016), including death from cardiovascular disease (CVD) (Gopinath et al. 2011; Eriksson et al. 2021), cerebrovascular disease (Fujino et al. 2007), and pregnancy disorders (Selander et al. 2019; Lissaker et al. 2021), including hearing dysfunction in children due to the mother being exposed during pregnancy (Selander et al. 2016). The impact of noise exposure on the cardiovascular system has also recently had a more detailed suggested mechanism describing an increase in levels of stress hormones and vascular oxidative stress which may lead to endothelial dysfunction and arterial hypertension (Munzel et al. 2018).

Job exposure from noise is historically studied in heavy industry settings but less so in day care or school environments (Eichwald and Scinicariello 2020).

Job-exposure matrices (JEMs) provide useful tools for developing historical exposure assessment where limited historical exposure information is available and for large studies where individual exposure assessment is not possible. Computerized JEMs were introduced in the late 1970s. The JEMs are matrices defined by a job axis and an exposure axis. An axis representing time period may form a third axis of the matrix. The cells in the JEM are usually based on measurements of different exposures in workplaces. Should no measurements exist, the vacant cells can be filled using any of the several different methods, for example, expert assessment usually aided by published literature and communication with the relevant occupational personnel. With respect to other noise JEMs, previous versions have less-well-defined levels of noise assessment and span over shorter time periods (Sjostrom et al. 2013), involve data from questionnaires (Schlaefer et al. 2009; Choi et al. 2012), or focus specific work environment sectors (Neitzel et al. 2018; Stokholm et al. 2020). The above-mentioned limitations mean that large variations in exposure could hide within the same assessment level, measurement data were lacking, or measurement-based assessments for a large portion of the work sector were limited. Thus, we, and others, lacked the ability to correctly analyze the relation between exposure and health outcomes for a major portion of the noise-exposed work environment. Therefore, we saw a need to create a JEM for noise including an expanded base of measurements, and a more detailed exposure-level assessment needed to be done spanning over a longer time period than our previous matrix (Sjostrom et al. 2013).).

## Methods

The JEM presented here was developed by 2 teams, each with an experienced occupational hygienist and an occupational safety engineer. Each pair assessed the exposure using measurements performed and reported by occupational hygienists, occupational safety engineers, or similar. The measurements were gathered over a time period from 1970 to 2014 and either originally included the LAeq(8h) or the LAeq(8h) was calculated from shorter measurements, provided the measurement targeted a regular task usually performed during a full workday. The collection of reports was done in 2008 and 2012 by contacting clinics working in the area of occupational health or occupational safety engineers and their submitted reports were added to our own material. The reports included personal measurements as well as stationary. The latter were included provided the measuring instrument was properly placed, and the worker exposed was also stationary during the measurement time. Reports with shorter than 8-h measurement time were included only if they were deemed to accurately depict the full 8-h exposure. Reports failing to meet the above criteria were not included. These data were incorporated into the matrix, at the appropriate time interval, for each corresponding job code. For some job families, for example, within heavy industry and childcare, 5 to 10 measurements could be available for a single time period. For others, like geologists and biologists, no measurements were available. The matrix uses the Nordic Occupational Classification system (NYK) definitions of job families and the 3-digit code on the job axis is based on the Nordic occupational code of 1985 (NYK-85/90), a modification of the 1983 version (Arbetsmarknadsstyrelsen 1983) based on the international ISCO-58. The exposure axis consists of an estimated average 8 h (TWA) noise-level classification for each job family in 1 of 5 different noise-level intervals, 1: <70 dB(A), 2: 70 to 74 dB(A), 3: 75 to 79 dB(A), 4: 80 to 84 dB(A), 5: 85(+) dB(A). These intervals were selected to evenly group assessed exposure up to the last that correspond to the occupational exposure limits for noise in Sweden. The TWA (LAeq, 8 h) for noise in Sweden is 85 dB(A) (8-h workday). Noise levels below 70 dB(A) are classified as low exposure in this JEM and levels >85 dB(A) are classified as high occupational exposure to noise. Data for the occurrence of peak exposure level (LpCpeak), of 135 dB(C) (the Swedish limit value), assessed with an instrument capable of obtaining a measurement within 50 μs were collected. However, the amount of data was low and thus the results did not live up to standards. We therefore chose not to include them in the JEM.

In our JEM, the most common noise level for the entire job family, consisting of 2 or more job titles, was selected. This included information on numbers of workers in different occupations (job title) in the Swedish workforce, obtained via the Swedish Central Bureau of Statistics (SCB), meaning that a job title with a higher number of workers has a larger influence on the noise level for the whole family. This meant that in a situation where job titles within a job family have different noise exposure assessments, the title with a higher number of workers directs the assessment for the job family as a group. Also, within a specific job title, there are many job tasks (welding, polishing, smithing) with high variability in exposure between tasks. Similarly, to the job families, the most common tasks have had the most influence on the noise level in the JEM.

### Validation analysis

The matrix was validated by 2 separate teams with an occupational hygienist and an occupational safety engineer in each team. The teams made separate exposure assessments for each job family based on measurements, supported by literature and/or professional experience regarding the specific occupation/family.

Assessments from each team were presented in a preliminary matrix and used for statistical analysis of relative position (RP) and relative concentration. The 2 suggested matrices were then combined by the 2 teams into a consensus matrix which is also used for similar statistical analysis.

### Statistical analysis

RP (Svensson 1997, 2000) is a measure that indicates if assessments are made differently between 2 assessors or pairs of assessors. Specifically, if assessments are lying on a straight line, from the lower-left to the upper-right corner, no systematic difference in assessments could be observed and the RP is 0. If, on the other hand, 1 assessor or 1 pair of assessors is consistently giving either lower or higher scores compared to the other assessor or pair of assessors, a systematic difference is observed, and the RP will not be 0. If a majority of assessments are gathered either in the lower-right or in the upper-left corner, the RP will be either negative or positive (see Supplementary Appendix 1).

Coefficient of monotonic agreement (MA) (Svensson 1997, 2000) is defined as the difference between the probabilities of ordered and disordered pairs of assessments. In situations when pairs of assessments are lying approximately on a straight line either from the lower-left to the upper-right corner or from the lower-right to the upper-left corner, the number of disordered pairs of assessments is either very few or very many. Hence, in these situations, MA is very high or very low, whereas if pairs of assessments are situated more or less evenly in lower-left corner, upper-left corner, upper-right corner, as well as lower-right corner, MA is approximately 0 since no clear pattern between assessments could be seen (see Supplementary Appendix 1).

## Results

A total of 864 measurements of noise exposure were gathered from 269 measurement reports. Each result was inserted into the JEM at the appropriate time period and corresponding to the appropriate job codes of the exposed persons. Some 2,000 gaps were filled by expert assessment, based on measurements of other time periods and supported by literature searches. The majority of these gaps pertained to low exposure, like office, environments.

The first row in the matrix contains the 3-digit job code followed by 5-yr periods and the letters N, L, and H. N denotes the assessed estimated average 8 h (TWA) noise level for the job family in the specific 5-yr period divided into categories 1 through 5, where 1: <70 dB(A), 2: 70 to 74 dB(A), 3: 75 to 79 dB(A), 4: 80 to 84 dB(A), 5: 85(+) dB(A). L denotes the lowest measured value found in the reports and H is the highest measured value, showing the width of the measurements.

The majority of the job families (81.9%) were assigned to the 3 lower levels of exposure to occupational noise. Office workers, administrators, legal workers, engineers, and advisors are examples of job families with low exposure. 10.9% was classified in the group with the second highest exposure (80 to 84 dB(A)) and 7.2 % was classified in the group with the highest exposure (85(+) dB(A)) to occupational noise. Job families with the highest exposure were, for example, miners, metal industry workers, and paper mill workers. The changes in the distribution of the different exposure levels during different time intervals from 1970 to 2014 are shown in Table 1.

**Table 1.** Distribution of job families across exposure levels during different time intervals.

Period/Exposure	1970 to 1974	1975 to 1979	1980 to 1984	1985 to 1989	1990 to 1994	1995 to 1999	2000 to 2004	2005 to 2009	2010 to 2014
Exp. lvl 1 <70 DB(A)	133	133	133	134	138	136	140	141	141
Exp. lvl 2 70 to 74 DB(A)	64	61	63	62	60	69	69	68	68
Exp. lvl 3 75 to 79 DB(A)	50	53	52	52	52	52	51	53	53
Exp. lvl 4 80 to 84 DB(A)	41	42	41	42	41	40	38	36	36
Exp. lvl 5 85(+) DB (A)	33	32	32	31	30	24	23	23	23

The table shows the number of job families for each level of assessed noise exposure over the time period 1970 to 2014.

As can also be seen in Table 1, the number of job codes with the 2 highest exposure levels, 4: 80 to 84 dB(A) and 5: 85(+) dB(A), is diminishing over the studied time period. Correspondingly, the number of job codes assigned to the 3 lowest exposure levels, 1: <70 dB(A), 2: 70 to 74 dB(A), and 3: 75 to 79 dB(A), is increasing.

A number of job codes were assigned a lower exposure level at a later time interval compared to previous time intervals (Table 2). The changes were due to either strong indicators in our gathered measurement data or clear technical advancements, in the respective occupational group, that would result in lower noise exposure. An example of such an advancement could be re-positioning of a worker to a control room instead of being close to heavy machinery with high noise levels. Other factors such as increased use of electronic and digital equipment instead of mechanical were also assessed to contribute to reduced exposure.

**Table 2.** Decreased noise exposure for some job families.

Time interval	Exposure change	Job family
70 to 74/75 to 79	5 to 4	755 Plumbers and pipe fitters
75 to 79/80 to 84	4 to 3	640 Bus and taxi drivers
80 to 84/85 to 89	2 to 1	661 Harbor masters
	3 to 2	801 Typesetters
	5 to 4	835 Plastic products makers
85 to 89/90 to 94	2 to 1	252 Computer operators 259 Computer processing workers (ADP) n.e.c. 691 Lighthouse and lock operators and harbor service assistants 801 Typesetters
	3 to 2	813 Glass and ceramics kilnmen 916 Pursers, stewards, stewardesses
	4 to 3	804 Bookbinders 812 Potters 827 Dairy workers 852 Tanners and fur dressers
	5 to 4	751 Toolmakers, machine-tool setters and operators
90 to 94/95 to 99	2 to 1	404 Forestry managers and supervisors
	3 to 2	401 Horticultural farmers 412 Livestock, dairy and poultry farm workers 431 Fishermen 631 Railway engine drivers 805 Photographic laboratory workers 809 Printing workers n.e.c. 828 Food processing and tobacco workers n.e.c.
	4 to 3	400 Working proprietors in agriculture and forestry 441 Loggers 641 Lorry and pickup drivers 829 Food processing and tobacco workers n.e.c. 861 Stationery engine and related equipment operators 871 Crane and hoist operators 872 Earth-moving and related machinery operators
	5 to 4	509 Mining and quarrying workers n.e.c. 803 Printing pressmen 825 Canning workers 833 Crushers, grinders and calender operators (chemical products) 839 Chemical processing workers n.e.c. 841 Paper-pulp preparers
95 to 99/00 to 04	2 to 1	769 Electrical and electronics workers n.e.c. 802 Printing and photo engravers
	3 to 2	640 Bus and taxi drivers 719 Truck and conveyor operators 762 Aircraft and vehicle electrician 763 Electrical machinery assemblers and repairmen
	4 to 3	758 Metal platers and coaters 803 Printing pressmen 873 Truck and conveyor operators
	5 to 4	754 Sheet-metal workers
00 to 04/05 to 09	2 to 1	629 Aircraft officers n.e.c.
	4 to 3	521 Ore dressers 599 Mining and quarrying workers n.e.c.

The table highlights the job families that have had their assessed noise exposure lowered over the study period 1970 to 2014.

Some job codes were assigned a higher exposure level at a later time level compared to an earlier one (Table 3). Several of these families pertain to teaching and childcare where several of our later measurement reports indicate a higher noise exposure than previously. A likely explanation for the increase is that the number of children in these groups has increased in size over time.

**Table 3.** Increase in noise exposure for some job families.

Time interval	Exposure change	Job family
70 to 74/75 to 79	2 to 3	431 Fishermen
80 to 84/85 to 89	2 to 3	34 Secondary education teachers (aesthetical-practical subjects)
85 to 89/90 to 94	3 to 4	76 Composers and musicians 153 Preschool teachers, Children’s nurses
90 to 94/95 to 99	1 to 2	32 Secondary education teachers (theoretical subjects) 33 Primary education teachers

The table highlights the job families that have had their assessed noise exposure increased over the study period 1970 to 2014.

In total, 2,889 assessments have been made based on 321 job families during 44 yr divided into 5-yr intervals, that is, 9 intervals. For noise exposure, the 2 pairs of assessors assessed the possible response alternatives <70, 70 to 74, 75 to 79, 80 to 84, 85(+) dB(A) according to the following percentage distribution: pair 1. *41.8; 19.6; 15.4; 13.2; 10.0* and pair 2. *41.7; 17.9; 18.3; 12.0; 10.1*. Comparisons of assessments were made between each pair and a consensus matrix. This matrix was derived from discussions between both pairs where they shared their experience and knowledge concerning noise exposure in different job families. Moreover, a comparison was also made between the 2 pairs. In total, 3 comparisons were made: 1st pair versus 2nd pair, 1st pair versus consensus assessments, and 2nd pair versus consensus assessments. The assessments from the consensus matrix are seen as independent compared to assessments made by the 1st and the 2nd pairs (see Supplementary Appendix 2).

### Noise exposure

There were systematic significant differences in RP, varied between 0.0191 and 0.0222, that is, both pairs assessed the job families as being more exposed in comparison to the consensus matrix. However, when both pairs met and discussed their assessments made for the job titles, new arguments and experiences were shared which led to minor changes to consensus. Interestingly, comparing the 2 pairs to each other revealed no systematic difference in RP = −0.003. The coefficient of MA, defined as the difference between the probabilities of ordered and disordered pairs of assessments, varied between 0.9656 and 0.9934. Hence, the agreement in ordering of paired ordinal classifications is very high (see Supplementary Appendix 1).

## Discussion

JEMs have some common limitations. One is the inability to account for the variability within the job family. Some job families contain several different job titles that may show considerable variations in noise levels between the specific occupations. The JEM in this paper provides a more detailed assessment of noise exposure for different job families compared to a JEM previously published by us (Sjostrom et al. 2013).

The major rationale for the update and extension of the previous JEM is the increased number of exposure levels introduced but also that it rests on 52% more measurements. In addition, the new JEM shows very small differences in expert assessments between the 2 expert groups used for validation.

Similar developments of JEMs exist based on a sizeable number of measurements and with detailed noise assessments but limited to a specific work sector (Neitzel et al. 2018; Stokholm et al. 2020). Other noise JEMs, and validation of these, based on questionnaires, have been described for German and American cohorts (Schlaefer et al. 2009; Choi et al. 2012).

## Results

When following changes in noise levels over time in the JEM, we find that 44 job families showed a decreased noise level over time. In contrast, 6 job families had an increased noise exposure over the study period. Interestingly, 4 out of 6 job families with increased exposure concerned childcare and teaching. This finding suggests an area in need of more studies regarding health outcomes related to noise exposure, especially for pregnant women (Selander et al. 2019).

### Previous JEM comparisons

Comparing the results to the previous version of noise JEM is complicated by the re-organization of noise assessment levels but the overall trend of the decreasing number of groups having a high level of exposure, as well as the corresponding increase in groups having low exposure, is more clearly seen in this extended JEM than in the earlier. However, the positional changes of groups previously clustered within “Medium exposure”, 75 to 85 dB(A), are only visible in the current version due to the more detailed assessments made.

Noise JEMs developed by other scientists, based on a large number of measurements and with detailed noise assessments (Neitzel et al. 2018; Stokholm et al. 2020), are recommended for use within the specific work sector they were developed for. Compared to those, the JEM presented in this paper would likely provide less sensitivity and accuracy in those settings. Our JEM, however, provides a broader view with assessments available for the whole of the Swedish workforce and may also be used for similar work environments.

### Validation

According to the validation analysis, there could be a slight risk of overestimation of noise exposure. However, discussion with Scandinavian colleagues, studying very similar work environments, and comparison to their assessments contradict this. Also, comparing the assessments of the 2 pairs of validation groups revealed no systematic difference in RP and found the agreement in ordering of paired ordinal classifications to be very high, thus, negating the initially perceived risk (see Supplementary Appendix 2). The discussions within the validation process were more often focused on the time period when a technical advancement or work environment legislation, resulting in changed noise exposure, had taken place but with high agreement on the resulting exposure level. The validation process and outcome, coupled with high reproducibility and numerous applications in studies hopefully, strengthens the confidence in this JEM.

### Strength and weaknesses

The noise JEM presented in this paper provides an increased sensitivity, compared to our previously published noise JEM, for studies related to noise exposure due to its more narrowly defined exposure groups. It also provides data over a longer time period than the previous version which affords the possibility to follow exposed persons in different job families over a whole working life. Also, the assessments made are for Swedish work environments with which we are very familiar through both clinical work and scientific studies. A factor that lowers the risk of misclassifications.

Even with the increased number of included measurements, a weakness of this study is the limited number of reports available. This is something we will try to amend in the future.

Another limitation of the JEM is the inability to account for the variability within the job family. The job families are dictated by the coding system used. Some job families contain a large number of different job titles with, sometimes, considerable variations in noise levels between the specific occupations. This is especially true for the job families designated by the coding system as “not elsewhere classified” where noise exposure assessment may span over 2 different exposure levels.

To mitigate this weakness, the most common noise level for the entire job family was selected. This also included an assessment of the number of workers in the different occupations, meaning that a job title with a lot of workers has a higher influence on the noise level for the whole family. The number of people working within a job title was obtained via the SCB. Also, for a specific job title, there are many job tasks with high variability in exposure between tasks. Similar to the case for the job families, the strategy has been that the estimated most common tasks have had the highest influence on the noise level in the JEM. Generic JEMs typically have a low sensitivity due to the number of jobs to be assessed and the often-unexpected circumstances in which occupational exposures can occur. Even with a satisfactory outcome of the validation analysis, one must keep practical restrictions in mind when applying the JEM. Included measurements of noise exposure are a mix of full-day measurements and, sometimes, extrapolated brief ones. The latter has the built-in uncertainty of possible over- or underestimation of exposure. Also, since the purpose was to estimate the most accurate exposure without mitigating factors as baseline that could then be improved upon, personal protective equipment has not been factored in when assessing exposure. Today, many of the workers with high exposure to occupational noise use ear protectors and are likely less exposed to occupational noise. However, this was less common at the earlier time points of the assessments and only very low-quality evidence exists that interventions to prevent occupational noise-induced hearing loss reduce risk (Tikka et al. 2017).

A number of job families were assessed to have a decrease in exposure, like printers and some industry workers. This is likely a benefit from technical improvements that not only result in lower noise from the equipment but also a dislocation due to a more automated process making it possible for the worker to monitor from an isolated control room. For job families with an increase in exposure, like teachers and childcare workers, we speculate that the changes are most likely due to an increase in the size of child groups.

The presented JEMs have already been used in several studies (Eriksson et al. 2018; Enoksson Wallas *et al.*, 2019; Wallas et al. 2018, 2019, 2020; Aarhus et al. 2020; Eriksson et al. 2021; Lissaker et al. 2021; Thacher et al. 2022) to study health effects from noise exposure such as cortisol levels, asthma, birth outcomes, and cardiovascular disease. We anticipate further use as an increasing number of epidemiological studies has shown health effects as a result of long-term exposure to noise (An et al. 2018; Kerns et al. 2018; Brauner et al. 2019; Liu et al. 2022; Wei et al. 2022).

## Conclusions

We have developed a JEM for occupational noise using Swedish data from 1970 to 2014 with a higher degree of sensitivity in assessed noise exposure compared to the previously existing version. The JEM has been validated with no systematic difference in RP and very high agreement in the ordering of paired ordinal classifications. Repeated application of the JEM, in epidemiological studies, has shown consistent results and has been a factor in yielding important findings.

## Supplementary Material

wxad074_suppl_Supplementary_Appendix_1

wxad074_suppl_Supplementary_Appendix_2

## Data Availability

The data, in the form of individual measurement reports, underlying this article cannot be shared publicly due to the privacy of individuals who participated in the measurement gathering. The data may be shared upon reasonable request to the corresponding author. The JEM can be made available to researchers within the field after contacting the first author via swejem@ki.se.
