# Supplementary material for: An updated job-exposure matrix for occupational noise: development and validation
Source: Ann Work Expo Health. 2023 Dec 9;68(2):146–54. doi: 10.1093/annweh/wxad074 (PMC10877457; doi:10.1093/annweh/wxad074)
Supplement: wxad074_suppl_Supplementary_Appendix_1 [file wxad074_suppl_supplementary_appendix_1.docx]

## An updated Job-Exposure Matrix for Occupational Noise: Development, and Validation

**Mattias Sjöström^1,2^*, Marie Lewné^1^, Magnus Alderling^1^,**

**Jenny Selander^1^ Per Gustavsson^1,2^**

1. *Institute of Environmental Medicine, Karolinska Institutet, Stockholm, Sweden.*
2. *Centre for Occupational and Environmental Medicine, Region Stockholm, Sweden.*

**Appendix 1**

**Statistical methods**

In order to assess the validity between exposure assessments performed by one pair, consisting of one occupational hygienists and one occupational safety engineer, compared to assessments performed by a second such pair, statistical measures presented by Svensson were used.[1-4] These measures are non-parametric and useful when assessments are done on an ordinal scale. The statistical measures reveal systematic differences between pairs of assessors by comparing the marginal distributions of assessments by each pair. Moreover, they reveal random differences by comparing augmented ranks between the pairs of assessors. The symbols X and Y in formulas presented below represents the first and second assessing pairs of experts respectively. The statistica relative position (RP) reveals if one pair of assessors systematically classify subjects lower or higher on the categorical scale compared to the other pair of assessors. The formula for relative position is:

$$RP=\sum_{v=1}^{m} \left( \hat{p}_{v}^{Y}\cdot\hat{P}_{v-1}^{X}-\hat{p}_{v}^{X}\cdot\hat{P}_{v-1}^{Y} \right)$$

where $\hat{p}_{v}^{X}$ and $\hat{p}_{v}^{Y}$ denote the $v$:th category relative frequencies and $\hat{P}_{v}^{X}$ and $\hat{P}_{v}^{Y}$ denote the cumulative $v$:th category relative frequencies of the assessments labeled $X$ and $Y$ respectively.

The valid range for this statistica is -1≤ RP ≤1. [1][3]

The statistica relative concentration (RC) reveals if one pair of assessors systematically use the center of the scale more often compared to the other pair. A requirement is that both pairs of raters had classified individuals to at least three different levels on the ordinal scale. The formula for relative concentration is:

$$RC= \frac{1}{min\left[ \hat{p}_{0}-\hat{p}_{0}^{2}, \hat{p}_{1}-\hat{p}_{1}^{2} \right]}\cdot\left[ \sum_{v=1}^{m} \hat{p}_{v}^{Y}\cdot\hat{P}_{v-1}^{X}\cdot\left( 1-\hat{P}_{v}^{X} \right)-\sum_{v=1}^{m} \hat{p}_{v}^{X}\cdot\hat{P}_{v-1}^{Y}\cdot\left( 1-\hat{P}_{v}^{Y} \right) \right]$$

Where

$$\hat{p}_{0}= \sum_{v=1}^{m} \hat{p}_{v}^{Y}\cdot\hat{P}_{v-1}^{X} ,\text{ }\hat{p}_{1}= \sum_{v=1}^{m} \hat{p}_{v}^{X}\cdot\hat{P}_{v-1}^{Y} \text{and }min \text{= minimum of} \hat{p}_{0}-\hat{p}_{0}^{2} \mathrm{and} \hat{p}_{1}-\hat{p}_{1}^{2}$$

The valid range for this statistica is -1≤ RC ≤1. [1][3]

The statistica relative rank variance (RV) reveals if the augmented ranks between pairs of assessors differ. Specifically, the ranking of individuals classified in a specific category by one of the raters is then determined by their internal order according to the classification of the other rater. The formula for relative rank variance is:

$$RV= \frac{6}{n^{3}}\sum_{i=1}^{m} \sum_{j=1}^{m} \left( \overline{R}_{ij}^{X}-\overline{R}_{ij}^{Y} \right)^{2}x_{ij}$$

where

$x_{ij}$ is the $\left( i,j \right)$: th cell frequency, $\overline{R}_{ij}^{X}\text{and}\text{ }\overline{R}_{ij}^{Y}\text{ }$ are the augmented mean rank values of the observations in the $\left( i,j \right)$: th cell for the two raters and $n$ is the number of individuals assessed.

The valid range for this statistica is 0≤ RV ≤1. [1][3]

The variances for RP, RC and RV respectively are estimated by jack-knife methods.

Another statistical measure proposed by Svensson is the coefficient of monotonic agreement (MA):

$$MA=1-2\cdot D$$

Where

$$D= \frac{\sum_{i=1}^{m_{1}} \sum_{j=1}^{m_{2}} x_{ij}\cdot\left( x_{ij}^{ul}+x_{ij}^{lr} \right)}{n\left( n-1 \right)-t}$$

Where

$x_{ij}$ is the $\left( i,j \right)$: th cell frequency and $x_{ij}^{ul}$ and $x_{ij}^{lr}$ are the upper-left and lower-right region frequencies relative to the $\left( i,j \right)$: th cell. The correction factor for observations that are tied for the same cells is:

$$t= \sum_{i=1}^{m_{1}} \sum_{j=1}^{m_{2}} x_{ij}\cdot\left( x_{ij}-1 \right)$$

The coefficient of monotonic agreement is a measure that defines the level of agreement in ordering of paired ordinal classifications relative to a total ordering, irrespective of the scaling and the marginal distributions. The valid range for this statistica is -1≤ MA ≤1. [2][4]

Relative operating characteristic curves (ROC:s) are created in a way of visualizing the systematic difference of assessments made by two pairs of assessors where the two sets of cumulative relative frequencies, $\hat{P}_{v}^{X}$ and $\hat{P}_{v}^{Y}$ for category $v$ are plotted against each other. The ROC curve coincides with the main diagonal in the case of no systematic disagreement, that is, equal marginal distributions. In case of a high absolute value of RP the curve is either below or above the main diagonal and in case of a high absolute value of RC the curve is S-shaped. [1]

The assessments regarding the noise level and the occurrence of peak levels were inputted into a sheet in Excel from the site: <http://www.oru.se/Akademier/Handelshogskolan/Kontakt-och-presentation/Personliga-sidor/Statistik/Elisabeth-Svensson/Svensoons-metod/Svenssons-metod---fri-programvara-och-dokumentation/> and the excel-macro returned, besides the crude percent agreement, the statistical measures relative position, relative concentration, relative rank variance and D along with their respective standard error and 95 % confidence intervals.

References

1. Svensson E and Holm S, 1994: Separation of systematic and random differences in ordinal rating scales. *Statistics in Medicine* **13**, 2437-53
2. Svensson E, 1997: A coefficient of agreement adjusted for bias in paired ordered categorical data. *Biometrical Journal* **39**, 643-57
3. Svensson E, 2000: Concordance between ratings using different scales for the same variable. *Statistics in Medicine* **19**, 3483-96
4. Comparison of the quality of assessments using continuous and discrete ordinal rating scales. *Biometrical Journal* **42**, 417-34
