# Supplementary material for: An updated job-exposure matrix for occupational noise: development and validation
Source: Ann Work Expo Health. 2023 Dec 9;68(2):146–54. doi: 10.1093/annweh/wxad074 (PMC10877457; doi:10.1093/annweh/wxad074)
Supplement: wxad074_suppl_Supplementary_Appendix_2 [file wxad074_suppl_supplementary_appendix_2.docx]

## An updated Job-Exposure Matrix for Occupational Noise: Development, and Validation

**Mattias Sjöström^1,2^*, Marie Lewné^1^, Magnus Alderling^1^,**

**Jenny Selander^1^ Per Gustavsson^1,2^**

1. *Institute of Environmental Medicine, Karolinska Institutet, Stockholm, Sweden.*
2. *Centre for Occupational and Environmental Medicine, Region Stockholm, Sweden.*

**Appendix 2**

**Acronyms:**

PA = Percent agreement, i.e. the percentage of judgements being judged equally by both pairs of judges

RP = Relative position, i.e. reveals if one pair of judges systematically classify subjects lower or higher on the categorical scale compared to the other pair of judges

RC = Relative concentration, i.e. reveals if one pair of judges systematically use the center of the scale more often compared to the other pair of judges

RV = Relative rank variance, i.e. reveals if the augmented ranks between pairs of assessors differ

D = This statistica is used to calculate MA, i.e. monotonic agreement calculated as 1-2*D. Monotonic agreement defines the level of agreement in ordering of paired ordinal classifications relative to a total ordering, irrespective of the scaling and the marginal distribution

SE = Standard error

Confidence Interval = The left and right column presents the lower and upper limit respectively to a 95 % confidence level
